# Data for properties of phosphoramide benzoazole oligonucleotides

**DOI:** 10.1016/j.dib.2026.112790

**Published:** 2026-04-18

**Authors:** Ivan I. Yushin, Victor M. Golyshev, Elizaveta E. Baranovskaya, Alexander A. Lomzov

**Affiliations:** a Laboratory of Structural Biology, Institute of Chemical Biology and Fundamental Medicine SB RAS, 8 Lavrentiev ave., Novosibirsk 630090, Russia; b Novosibirsk State University, 1 Pirogova st., Novosibirsk 630090, Russia

**Keywords:** Phosphoramide benzimidazole oligonucleotides, ABAO, Structure, Thermodynamics, Molecular dynamics

## Abstract

This article presents new data on the physicochemical properties of phosphoramide benzoazole oligonucleotides (PABAOs) bearing *N*-benzothiazole, *N*-benzimidazole, and *N*-benzoxazole phosphoramidate groups. The data include: 1) temperature series of circular dichroism and UV–Vis spectra of the native and modified duplexes; 2) data from singular value decomposition analysis of UV–Vis spectra used for pK_a_ value determination of the phosphoramidate *N*-benzothiazole moiety; 3) values of thermodynamic parameters (changes in enthalpy, entropy, Gibbs free energy and melting temperature) of DNA/DNA and PABAO/DNA duplexes; 4) partial atomic charges of the phosphoramidate *N*-benzazole moiety; 5) structures of DNA/DNA and PABAO/DNA duplexes obtained using k-means cluster analysis of molecular dynamics trajectories. The data can be used for rational design of PABAOs as probes for various applications and in basic research. The data are associated with the manuscript “Properties of phosphoramide benzoazole oligonucleotides (PABAOs). III. Structure and hybridization efficiency of N-benzothiazole derivatives”.

Specifications Table

**Table:** 

Subject	Biology
Specific subject area	Paper contain raw data on physicochemical properties of PABAOs in comparison with native DNA
Type of data	Tables in csv format, molecules structure in files of pdb format Raw data of circular dichroism and partial atomic charges, Analyzed data of pKa determination and thermodynamic parameters, Processed data of molecular dynamics simulation.
Data collection	To determine thermodynamic parameters and pKa Cary3500 spectrophotometer (Agilent, USA) was used. Thermodynamic parameters were obtained by non-linear fitting of UV-melting curves via the two-state model. The pKa values were calculated using singular value decomposition (SVD) method and using the Henderson-Hasselbalch equation. Circular dichroism (CD) spectra were recorded on Jasco J-600 (Jasco, Japan) spectropolarimeter. Molecular dynamics simulations were performed using AMBER23 and AMBERTOOLS23 software.
Data source location	Laboratory of Structural Biology, Institute of Chemical Biology and Fundamental Medicine SB RAS, 8 Lavrentiev ave., Novosibirsk 630,090, Russia*.*
Data accessibility	Repository name: Mendeley data Data identification number: doi: 10.17632/8vwtnzm2fy.2 Direct URL to data: https://data.mendeley.com/datasets/8vwtnzm2fy/2
Related research article	Properties of phosphoramide benzoazole oligonucleotides (PABAOs). III. Structure and hybridization efficiency of N-benzothiazole derivatives. I.I. Yushin, V.M. Golyshev, E.E. Baranovskaya, A.A. Lomzov. Biochem. Biophys. Res. Commun. (2025) 153,170, DOI: 10.1016/j.bbrc.2025.153170

## 1 Value of the Data

These data are valuable for researchers performing comparative analysis of phosphate-modified nucleic acids. They can be used by scientists interested in the synthesis, purification, and application of nucleic acid derivatives and analogues. These results advance the understanding of the properties of phosphate-modified oligonucleotides. The dataset will be useful to researchers in molecular biology, biotechnology, and the biomedical applications of nucleic acids.

## 2 Background

Modified oligonucleotides serve as a cornerstone of innovation in therapeutic and diagnostic applications. Chemical modifications to the nucleobase, sugar, or phosphate backbone enable the precise tailoring of their physicochemical and biological properties. Among recent developments, phosphoramidate benzoazole oligonucleotides (PABAOs) have emerged as a promising class of derivatives [[1], [2]]. These modifications significantly influence oligonucleotide charge, hydrophobicity, and hybridization efficiency, leading to improved nuclease stability and enhanced specificity in nucleic acid detection [[2], [3], [4]]. Their synthesis is efficiently achieved via the Staudinger reaction, a method that facilitates high-yield, site-specific incorporation of diverse functional groups directly during automated solid-phase synthesis [5,6]. By varying the structure of the azide reactant, the properties of the resulting oligonucleotides can be finely modulated, establishing this methodology as a versatile tool for both applied nucleic acid engineering and fundamental biophysical research. Expanding upon prior studies [[3], [4], [5]], this study delivers a comprehensive and detailed analysis of the spectral signatures of PABAOs, the thermodynamic stability of their DNA duplexes, and the structural consequences of the modification [1].

## 3 Data Description

This dataset [7] has five items:

1) temperature series of circular dichroism and UV–Vis spectra of the native and modified duplexes It includes three csv files for the duplexes of 8, 10, and 12 bp in length with the CD spectra at 5 and 95 °C. CD spectra of the modified complexes at different temperatures in the range 5 and 95 °C. (folders: CD and CD_melting). The data can be used for quantitative analysis and comparison of the CD spectra and secondary structures of oligonucleotides and their complexes.

Folder CD contains files 8_CD.csv, 10_CD.csv, and 12_CD.csv with CD spectra of octa-, deca-, and dodecamer complexes.

Folder CD_melting contains files M8_N8X.csv, M8X_N8.csv, M10_N10X.csv, M10X_N10.csv, M12_N12X.csv, and M12X_N12.csv with CD spectra of octa-, deca-, and dodecamer complexes =at different temperatures.

2) UV–Vis absorption spectra of the octa-, deca-, and dodecamer in water and hexathymidylate 5'-T*TTTTT-3'bearing *N*-benzothiazole modification in buffer solutions with varying pH values. Data of singular value decomposition analysis of UV–Vis spectra (folders: UV–Vis_thiazole and pKa_SVD_analysis). The data can be used for optical spectra analysis of oligonucleotides and benzothiazole moieties at different pH values.

Folder UV–Vis_thiazole contains file UV–Vis_thiazole.csv with UV–Vis absorption spectra of modified octa-, deca-, and dodecamers.

Folder pKa_SVD_analysis contains files: alpha_experimental.csv – calculated experimental fractions of different protonation forms; alpha_theoretical.csv - calculated theoretical fractions of different protonation forms; thio_component_experimental.csv – calculated experimental fractions of components obtained via SVD analysis at different pH values; thio_component_theoretical.csv - calculated theoretical fractions of components at different pH values; thio_normalized_267nm.csv – experimental UV–Vis spectra normalized at 267 nm; thio_pKa_components.csv - calculated experimental absorption spectra of components at different wavelength; thio_pKa_source.csv – experimental UV–Vis absorption spectra in buffer solutions with different pH values.

3) Values of thermodynamic parameters (changes in enthalpy, entropy, Gibbs free energy and melting temperature) of DNA/DNA and PABAO/DNA duplexes (folder: thermodynamic_parameters). The data can be used for detailed thermodynamic analysis of nucleic acids and their derivatives, including the development of predictive models.

Folder thermodynamic parameters contains file thermodynamic_parameters.csv with values of thermodynamic parameters (changes in enthalpy, entropy, Gibbs free energy and melting temperature) of DNA/DNA and PABAO/DNA duplexes for *N*-benzothiazole, *N*-benzimidazole, and *N*-benzoxazole derivatives.

4) Partial atomic charges of the phosphoramidate *N*-benzazole moiety (folder: patial_charges). The data are useful for MD simulation of N-benzoazole nucleic acid derivatives.

Folder contains files: BN_charges.dat, BN_conformers.sdf, BO_charges.dat, BO_conformers.sdf, BS_charges.dat, BS_conformers.sdf, NH_charges.dat, NH_conformers.sdf, OH_charges.dat, OH_conformers.sdf, SH_charges.dat, and SH_conformers.sdf. Files with dat extension contain partial atomic charge in last column. Files with sdf extension contain are the optimized geometry structures. BN(NH), BO(OH), and BS(SH) in file names denotes *N*-benzimidazole, *N*-benzoxazole, and *N*-benzothiazole phosphoramidate derivatives at deprotonated(protonated) states.

5) Structures of DNA/DNA and PABAO/DNA duplexes obtained using k-means cluster analysis (most representative structure) of molecular dynamics trajectories (folder: molecular_dynamics_structures). The data can be used for structural analysis of nucleic acids and their derivatives.

Folder contains subfolders Native, BN, BO, BS, NH, OH, SH with data for native duplexes, and modified with deprotonated or protonated modifications. Every folder for modified complexes contains three subfolder 8, 10, and 12 for complexes of corresponding length. In every folder 8 files for duplexes in both chains with different combination of stereoisomers are presented.

## 4 Experimental Design, Materials and Methods

Oligonucleotides with modified N-benzoazole phosphate groups were synthesized and purified as described previously [1].

UV–Vis spectra (range 200 to 800 nm) were recorded using the Cary3500 spectrophotometer (Agilent, USA) and the quartz cuvette with an optical path length of 10 mm.

The pKa values were calculated using SVD method and using the Henderson-Hasselbalch equation.

CD spectra (range 200–360 nm) were recorded on JASCO J600 spectrophotometer (JASCO, Japan) with the use of quartz cell of 1 cm light path length in the temperature range of 5–95 °C. Temperature were controlled by the Huber-CC3 water bath (Huber, Germany). Presented CD spectra are averaging of 5 replicates.

UV-melting curves were obtained on Cary3500 spectrophotometer (Agilent, USA) using 2 mm optical path length quartz cuvettes as described in [1]. Values of thermodynamic parameters were determined by non-linear fitting and two-state model approximation [1].

Partial atomic charges were calculated using PyRESP software based on single point calculations of optimized structures (HF/6–31G//B3LYP/6–31G++(3df,3pd)) obtained in Gaussian’09.

MD simulations were performed in AMBER23 software package as described in [1]. Obtained 100 ns trajectories were analysed by k-means clustering.

## Limitations

Not applicable.

## Ethics Statement

All authors have read and follow the ethical requirements for publication in Data in Brief and confirming that the current work does not involve human subjects, animal experiments, or any data collected from social media platforms.

## Credit Author Statement

**Ivan I. Yushin:** Methodology, Data curation, Original draft preparation, Investigation, Writing, Writing- Reviewing and Editing; **Victor M. Golyshev:** Methodology, Data curation, Writing, Writing- Reviewing and Editing; **Elizaveta E. Baranovskaya**: Methodology, Data curation, Writing- Reviewing and Editing; **Alexander A. Lomzov**: Conceptualization, Methodology, Original draft preparation, Supervision, Writing- Reviewing and Editing.

## Data Availability

Mendeley DataData on properties of phosphoramide benzoazole oligonucleotides (Original data).
